# Supplementary material for: The Association between Non-Alcoholic Fatty Liver Disease (NAFLD) and Advanced Fibrosis with Serological Vitamin B12 Markers: Results from the NHANES 1999–2004
Source: Nutrients. 2022 Mar 14;14(6):1224. doi: 10.3390/nu14061224 (PMC8948655; doi:10.3390/nu14061224)
Supplement: Supplementary file 1 [file nutrients-14-01224-s001.zip › nutrients-1581050-supplementary.pdf]

Supplementary

Table S1. The association of MMA and HCY with fibrosis diagnosed by NFS, FIB-4, APRI or combinations of them in participants with or without NAFLD defined by FLI, USFLI or combinations of both after adjustment.

		NAFLD_FLI	NAFLD_USFLI	NAFLD	Total
N (%)		3639 (42.4)	1283 (29.4)	3835 (44.2)	8397 (100)
	RBC-folate	2.28 (1.47, 3.54)**	1.62 (1.06, 2.49)*	2.24 (1.57, 3.18)**	
Fibrosis_NFS	MMA	1.47 (0.98, 2.19)	1.74 (0.99, 3.06)	1.49 (1.06, 2.09)*	1.38 (1.05, 1.81)*
	HCY	1.49 (0.75, 2.98)	2.27 (0.63, 8.18)	1.66 (0.88, 3.11)	1.82 (0.99, 3.34)
Fibrosis_FIB4	MMA	1.37 (0.87, 2.15)	1.25 (0.51, 3.04)	1.24 (0.77, 2.00)	1.40 (1.00, 1.97)
	HCY	2.53 (1.01, 6.35)*	2.62 (0.50, 13.68)	2.35 (0.96, 5.76)	2.67 (1.49, 4.76)*
Fibrosis_APRI	MMA	1.03 (0.35, 3.01)	0.36 (0.09, 1.41)	1.06 (0.38, 2.95)	0.90 (0.47, 1.76)
	HCY	2.83 (0.70, 11.42)	1.00 (0.09, 11.42)	2.87 (0.72, 11.45)	5.19 (1.77, 15.21)*
Fibrosis	MMA	1.38 (0.97, 1.96)	1.32 (0.87, 1.98)	1.37 (1.01, 1.84)*	1.40 (1.07, 1.83)*
	HCY	1.79 (0.95, 3.39)	1.91 (0.64, 5.69)	1.90 (1.07, 3.35)*	2.78 (1.49, 5.19)*

Fibrosis was defined by NFS, FIB4, APRI or combinations of these three indices (indicated by “fibrosis” in the table), and NAFLD was diagnosed by FLI, USFLI or

combinations of them (shown by “NAFLD” in the table). Associations between MMA or HCY and fibrosis were investigated within each defined category of NAFLD.

Adjustment: age+sex+races+smoking+BMI+diabetes+hypertension+dyscholestrolemia+kidney function+ log-transformed-VitB12+log-transformed-serum folate.

Values were presented as the weighted proportion (%) and odds ratio (95% CI), **P≤0.001, *P<0.05.

FLI, fatty Liver Index; USFLI, U.S. Fatty Liver Index; MMA, methylmalonic acid; HCY, homocysteine; NFS, NAFLD fibrosis score; FIB-4, Fibrosis-4 index; APRI, AST-to-platelet ratio index; NAFLD, non-alcoholic fatty liver disease.

Table S2: Characteristics of participants with normal or abnormal MMA or HCY in participants from NHANES 1999–2004.

	**MMA (nmol/L)**			**HCY (μmol/L)**		
	MMA <250	MMA ≥250	P	HCY<15	HCY≥15	P
N (%)	7470 (90.3)	927 (9.7)		7904 (95.5)	493 (4.5)	
FLI	50.0 (48.7, 51.3)	52.0 (49.8, 54.2)	0.1112	50.0 (48.7, 51.2)	55.0 (51.1, 58.9)	0.0206
USFLI	23.2 (22.0, 24.3)	28.5 (25.6, 31.4)	<0.001	23.4 (22.2, 24.6)	30.3 (24.7, 35.8)	0.0136
NFS	-2.32 (-2.38, -2.27)	-1.44 (-1.54, -1.33)	<0.001	-2.29 (-2.34, -2.24)	-1.16 (-1.31, -1.02)	<0.001
FIB-4	0.98 (0.96, 1.00)	1.39 (1.34, 1.45)	<0.001	0.99 (0.98, 1.01)	1.572 (1.47, 1.68)	<0.001
APRI	0.286 (0.281, 0.290)	0.295 (0.283, 0.307)	0.1884	0.285 (0.280, 0.289)	0.328 (0.291, 0.366)	0.0264
NAFLD (%)	44	46.1	0.2524	43.9	50.5	0.0113
fibrosis (%)	3.7	12.5	<0.001	3.9	19.2	<0.001

MMA>=250 nmo/L and HCY >=15umol/L were used to define abnormal MMA or HCY levels. FLI, fatty Liver Index; USFLI, U.S. Fatty Liver Index; MMA, methylmalonic acid; HCY, homocysteine; NFS, NAFLD fibrosis score; FIB-4, Fibrosis-4 index; APRI, AST-to-platelet ratio index; NAFLD, non-alcoholic fatty liver disease.

Table S3. The association between MMA, HCY or RBC-folate and metabolic disorders (including diabetes, hypertension and dyscholestrolemia) in participants from NHANES 1999–2004.

	(OR)	Diabetes	Hypertension	Dyscholestrolemia
Model 1	MMA	1.74 (1.40, 2.17)**	2.43 (2.08, 2.83)**	1.14 (1.04, 1.26)*
	HCY	2.80 (2.30, 3.41)**	5.49 (4.49, 6.70)**	1.26 (1.07, 1.48)*
	RBC-folate	1.97 (1.57, 2.47)**	2.36 (2.01, 2.76)**	1.34 (1.16, 1.54)**
Model 2	MMA	1.10 (0.83, 1.46)	1.33 (1.13, 1.57)**	0.96 (0.86, 1.08)
	HCY	1.09 (0.81, 1.47)	2.01 (1.52, 2.67)**	0.97 (0.80, 1.17)
	RBC-folate	1.37 (1.09, 1.72)*	1.20 (1.02, 1.42)*	1.10 (0.95, 1.27)
Model 3	MMA	1.01 (0.74, 1.37)	1.25 (1.04, 1.49)*	1.01 (0.90, 1.12)
	HCY	0.85 (0.60, 1.19)	1.69 (1.26, 2.25)**	1.00 (0.83, 1.22)
	RBC-folate	1.29 (1.04, 1.60)*	1.11 (0.93, 1.33)	1.06 (0.91, 1.22)
Model 4	MMA	1.07 (0.80, 1.42)	1.23 (1.01, 1.49)*	0.99 (0.86, 1.13)
	HCY	0.90 (0.63, 1.29)	1.80 (1.28, 2.53)**	0.95 (0.74, 1.22)
	RBC-folate	1.38 (1.04, 1.82)*	1.17 (0.94, 1.45)	1.14 (0.95, 1.37)

Logistic regression was used to detect the odds ratio (95% CI) for metabolic disorders of MMA, HCY and RBC-folate. **$P \leq 0.001$, *$P < 0.05$

Model 1: unadjusted;

Model 2: age + sex + races + smoking;

Model 3: model 2 + BMI + diabetes + hypertension + dyscholestrolemia + kidney function;

Model 4: model 3 + log-transformed-VitB12 + log-transformed- serum folate;

MMA, methylmalonic acid; HCY, homocysteine; RBC-folate: folate in red blood cells.
